# Thermal Stability of Encapsulated Carbon-Based Multiporous-Layered-Electrode Perovskite Solar Cells Extended to Over 5000 h at 85 °C

**DOI:** 10.3390/ma17123002

**Published:** 2024-06-19

**Authors:** Ryuki Tsuji, Yuuma Nagano, Kota Oishi, Eiji Kobayashi, Seigo Ito

**Affiliations:** 1 Department of Materials and Synchrotron Radiation Engineering, Graduate School of Engineering, University of Hyogo, 2167 Shosha, Himeji 671-2280, Hyogo, Japan; 2 Department of Materials Science, Institute of Pure and Applied Sciences, University of Tsukuba, 1-1-1 Tennodai, Tsukuba 305-8573, Ibaraki, Japan; 3 Kishu Giken Kogyo Co., Ltd., 446 Nunohiki, Wakayama 641-0015, Wakayama, Japan

**Keywords:** carbon electrode, printable mesoscopic, thermal durability, encapsulation, sealing

## Abstract

The key to the practical application of organometal–halide crystals perovskite solar cells (PSCs) is to achieve thermal stability through robust encapsulation. This paper presents a method to significantly extend the thermal stability lifetime of perovskite solar cells to over 5000 h at 85 °C by demonstrating an optimal combination of encapsulation methods and perovskite composition for carbon-based multiporous-layered-electrode (MPLE)-PSCs. We fabricated four types of MPLE-PSCs using two encapsulation structures (over- and side-sealing with thermoplastic resin films) and two perovskite compositions ((5-AVA)_x_(methylammonium (MA))_1−x_PbI_3_ and (formamidinium (FA))_0.9_Cs_0.1_PbI_3_), and analyzed the 85 °C thermal stability followed by the ISOS-D-2 protocol. Without encapsulation, FA_0.9_Cs_0.1_PbI_3_ exhibited higher thermal stability than (5-AVA)_x_(MA)_1−x_PbI_3_. However, encapsulation reversed the phenomenon (that of (5-AVA)_x_(MA)_1−x_PbI_3_ became stronger). The combination of the (5-AVA)_x_(MA)_1−x_PbI_3_ perovskite absorber and over-sealing encapsulation effectively suppressed the thermal degradation, resulting in a PCE value of 91.2% of the initial value after 5072 h. On the other hand, another combination (side-sealing on (5-AVA)_x_(MA)_1−x_PbI_3_ and over- and side-sealing on FA_0.9_Cs_0.1_PbI_3_) resulted in decreased stability. The FACs-based perovskite was decomposed from these degradation mechanisms by the condensation reaction between FA and carbon. For side-sealing, the space between the cell and the encapsulant was estimated to contain approximately 1,260,000 times more H_2_O than in over-sealing, which catalyzed the degradation of the perovskite crystals. Our results demonstrate that MA-based PSCs, which are generally considered to be thermally sensitive, can significantly extend their thermal stability after proper encapsulation. Therefore, we emphasize that finding the appropriate combination of encapsulation technique and perovskite composition is quite important to achieve further device stability.

## 1. Introduction

Society is demanding renewable energy sources to replace CO_2_-emitting energy. As an alternative, perovskite solar cells (PSCs) have attracted much attention. PSCs were invented by Prof. T. Miyasaka and his group in 2009 [1]. As of May 2024, a power conversion efficiency (PCE) of 26.07% (certified 25.8%) for PSCs has been achieved by Prof. H. Zhou and his group [2]. This is close to the world’s highest efficiency of 26.81% for widely used Si solar cells [3]. In addition, the PSCs can be fabricated using only low-cost processes such as coating and printing. Therefore, they have the potential to be a cheaper source of energy than existing solar cells.

However, to make PSCs more viable than existing solar cells, it is necessary to reduce material and manufacturing costs and improve durability [4]. Typical PSCs use expensive organic charge transport materials (e.g., Spiro-MeOTAD, fullerene C_60_, and PEDOT:PSS) and precious metals as back contacts (e.g., Au and Ag). These electrodes are formed by vacuum evaporation, and the equipment cost can be high. In addition, it has been reported that Au and Ag electrodes are easily degraded by metal migration under operating conditions [5,6]. It has also been reported that perovskite crystals are easily decomposed by moisture and oxygen in the air [7].

To solve these problems of PSCs, it is important to (1) replace unstable materials with stable materials and (2) establish an appropriate encapsulation technology.

Regarding point (1), to utilize stable materials, carbon-based fully printable mesoscopic PSCs were developed by Prof. H. Han and his group in 2013, replacing expensive and unstable organic materials and metal electrodes with carbon electrodes [8,9]. These PSCs used a three-layer mesoscopic structure of mesoporous TiO_2_ (m-TiO_2_) as the electron transport layer (0.5 μm), m-ZrO_2_ as the insulating layer (1–3 μm), and porous carbon as both the hole transport layer and back contact (>10 μm). This structure is based on the prototype monolithic dye-sensitized solar cells (DSSCs) developed by Prof. M Grätzel and his group in 1996 [10]. This monolithic structure will be referred to as a “multiporous-layered-electrode (MPLE) structure” in this manuscript. The PCE of MPLE-PSCs is lower than thin film (TF)-PSCs with metal electrodes. In the published work on MPLE-PSCs, the PCE is mostly 10~19% [11,12,13], with a maximum of 22.2% [14]. On the other hand, in the published work on TF-PSCs, the PCE can be over 20% [15], with a maximum of 26.07% [2]. The low PCE of MPLE-PSCs can be improved by strategies such as enhanced hole transport capability, energy level matching, and dense packing of larger perovskite crystals within the porous layer. For more information, see the excellent review articles [16,17,18].

However, the most important features of MPLE-PSCs are their low manufacturing cost and excellent stability [19]. This is because the thick carbon electrodes of MPLE-PSCs effectively prevent water and oxygen from entering the cell [20,21]. In fact, many of the published papers reported results of high stability in light, maximum power point tracking (MPPT), heat, and moisture. G. Grancini et al. reported light stability of one year (>10,000 h) for MPLE-PSCs with 2D/3D perovskite and AVAI as an additive [22]. In addition, A. Mei et al. reported that MAPbI_3_-based MPLE-PSCs with (5-AVA) iodide as an additive passed the MPPT test for >9000 h [23]. Furthermore, the authors reported that encapsulated MPLE-PSCs with similar (5-AVA)_x_MA_1−x_PbI_3_ passed the dump-heat test (85 °C/85%RH, International Summit on Organic Photovoltaic Stability (ISOS)-D-3 protocol) for >3000 h [20].

Regarding point (2), to commercialize PSCs, a proper encapsulation technology is required to withstand harsh outdoor conditions [24]. L. Shi et al. reported that encapsulated TF-PSCs using Cs_0.05_FA_0.8_MA_0.15_Pb(I_0.85_Br_0.15_)_3_ perovskite with a polyisobutylene-based polymer blanket and cover glass sheets passed a dump-heat test of >1800 h and a humidity freeze test of >75 cycles [25]. Their results show that mixed cation cells containing Cs have the highest stability. In addition, the encapsulation method was also examined, showing that a wide blanket is superior to an edge seal. Therefore, the combination of the perovskite crystal composition, encapsulant, and encapsulation method must be optimized to effectively prevent perovskite crystal degradation [26].

Similarly, encapsulants and sealing methods have been also investigated for MPLE-PSCs. Z. Fu et al. reported that MPLE-PSC submodules (10 × 10 cm) encapsulated with polyurethane and glass sheets maintained 97.52% of their initial efficiency after 2136 h under outdoor conditions (−10 to 35 °C) [27]. They used an over-sealing method that covered the entire surface of the solar cells. In a previous study by the authors, over- and side-sealing methods were compared for MPLE-PSCs using UV-curing glue [28,29]. When UV-curing glue was used as the encapsulant, the side-sealing method showed better stability than the over-sealing method. Therefore, based on the results of these studies, appropriate sealing methods should be used depending on the type of encapsulant.

**Figure 1:**
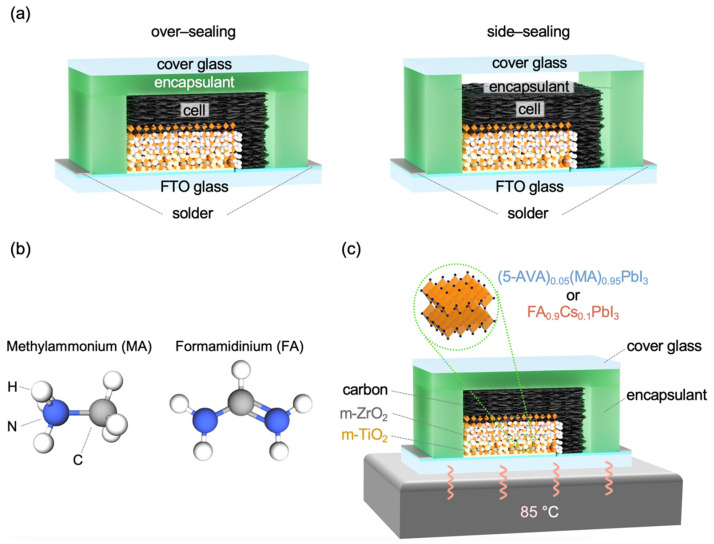
(**a**) Schematic encapsulation diagram of over- and side-sealing multiporous-layered-electrode perovskite solar cells (MPLE-PSCs). (**b**) Chemical structures of methylammonium (MA) and formamidinium (FA). (**c**) Schematic illustration of thermal stability test for encapsulated MPLE-PSCs.

Based on these previous studies, we have attempted here to optimize the appropriate combination of encapsulation method and perovskite crystal compositions for the commercialization of MPLE-PSCs. The authors recently reported that MPLE-PSC modules encapsulated with hot-melt ionomer films have stability equivalent to 20 years of outdoor use [20]. In the previous report with hot-melt ionomer films, only the over-sealing method was used, and the side-sealing method was not investigated. Therefore, this study compared the effectiveness of over- and side-sealing methods using hot-melt ionomer films (Figure 1a).

The perovskite crystal composition of MPLE-PSC was also compared between methylammonium (MA)-based and formamidinium cesium (FA)-based perovskites (Figure 1b). Due to differences in the molecular size and bonding strength, MA perovskite has lower thermal stability than FACs perovskite [30]. However, MA-based perovskites are widely used in MPLE-PSCs and have demonstrated high stability, making them mainstream. On the other hand, high-efficiency FACs-based MPLE-PSCs have been successful in only a few groups, and their potential is still unknown [31,32,33,34]. Therefore, we also addressed the comparison between MA and FACs perovskites.

In this study, to properly evaluate the thermal stability of MPLE-PSCs with MA and FACs perovskites, thermal stability tests were performed at 85 °C according to the ISOS-D-2 protocol [35]. The standard thermal stability test for solar cells is typically performed in the range of −40 to +85 °C. In this study, only the high-temperature side (85 °C) was focused on first because perovskite crystals, especially MA perovskite, are known to decompose when exposed to high temperatures (Figure 1c).

## 2. Experimental Section

### 2.1. Materials

All commercially available raw materials were used as is without purification. The materials used are as follows: titanium diisopropoxide bis (acetylacetonate) (75 wt.% in isopropanol, Sigma-Aldrich, St. Louis, MO, USA), titanium dioxide (TiO_2_) paste (PST-30NRD, JGC Catalysts and Chemicals, Kawasaki, Japan), zirconium dioxide (ZrO_2_) paste (Zr-Nanoxide ZT/SP, Solaronix SA, Aubonne, Switzerland), α-terpineol (Kanto Chemical, Tokyo, Japan), lead iodide (PbI_2_, 99.99%, Tokyo Chemical Industry, Tokyo, Japan), methylammonium iodide (MAI, 98.0%, Tokyo Chemical Industry, Japan), 5-ammonium valeric acid iodide (5-AVAI, Greatcell Solar, Queanbeyan, Australia), γ-butyrolactone (GBL, electrochemical grade, Kanto Chemical, Japan), formamidine hydroiodide (FAI, 99.99%, Tokyo Chemical Industry, Japan), cesium iodide (CsI, 99.0%, Tokyo Chemical Industry, Japan), N,N-dimethylformamide (DMF, FUJIFILM Wako Pure Chemical Corporation, Osaka, Japan), dimethyl sulfoxide (DMSO, FUJIFILM Wako Pure Chemical Corporation), fluorine-doped tin oxide (FTO) glass (TEC-15, Nippon Sheet Glass-Pilkington, Tokyo, Japan), thermoplastic resin films consisting of an ionomer-based material (KuranSeal-ES, Kurabo Industries Ltd., Osaka, Japan), and cover glass (ASLAB Slide Glass, AS ONE Corporation, Osaka, Japan). Preparation of the perovskite precursor solution was performed in a glove box (N_2_ gas, the dew point was −30 to −20 °C). The 1.2 M (5-AVA)_0.05_(MA)_0.95_PbI_3_ perovskite precursor was obtained by dissolving 0.2776 g PbI_2_, 0.0906 g MAI, and 0.0074 g 5-AVAI in 500 μL GBL, and then stirred at 70 °C overnight. The 1.0 M FA_0.9_Cs_0.1_PbI_3_ perovskite precursor solution was obtained by dissolving 0.2305 g PbI_2_, 0.0774 g FAI, and 0.0130 g CsI in 500 μL DMF/DMSO (=4:1, *v*/*v*), and then stirred at 50 °C overnight.

### 2.2. Device Fabrication

The device used in this work was fabricated according to the procedures previously reported in the literature [36,37,38,39,40,41]. All device fabrication processes were performed under ambient air conditions. The FTO glass substrate (100 × 60 mm) was separated to be small solar cells by laser etching instrument. The etched substrate was ultrasonically cleaned with a detergent solution (1 wt.%, white 7-AL, Yuai Kasei, Amagasaki, Japan) and ethanol for 15 min, respectively. The compact TiO_2_ (c-TiO_2_) layer was deposited on substrates by spray pyrolysis deposition (SPD) with a 0.66 mL titanium diisopropoxide bis (acetylacetonate) solution diluted in 22.5 mL ethanol (1:34 volume ratio) on a hot plate at 500 °C. The 0.5 μm mesoporous TiO_2_ (m-TiO_2_) layer was screen printed using the TiO_2_ paste. The 1.0 to 1.5 μm mesoporous ZrO_2_ (m-ZrO_2_) layer was screen printed using the ZrO_2_ paste. Then, the TiO_2_ and ZrO_2_ layers were annealed at 500 °C for 1 h on a hot plate. After cooling down, the 10 to 20 μm carbon layer was screen printed using carbon paste on the porous ZrO_2_ layer. The carbon layer was annealed at 400 °C for 1 h on a hot plate. Then, the substrates were cooled to room temperature and separated into single cells. The multiporous-layered electrodes were sintered at 400 °C for 50 min on a hot plate before applying the perovskite precursor solution. Once the substrate had cooled, electrical metal contacts were attached to both external contacts of the cell substrate by ultrasonic soldering. The area around the porous electrodes was masked with heat-resistant polyimide tape so that the two types of perovskite solutions ((5-AVA)_0.05_(MA)_0.95_PbI_3_ or FA_0.9_Cs_0.1_PbI_3_) filled in the porous electrodes, as described below.

(5-AVA)_0.05_(MA)_0.95_PbI_3_ perovskite: 4.0 μL of the precursor solution was supplied to the porous electrodes by drop-casting. The devices were kept with a petri dish cover at room temperature for 30 min, and then transferred to a 50 °C hot plate with a petri dish cover and kept for 30 min. Then, the petri dish cover was removed and the device was dried at 50 °C for 1 h.

FA_0.9_Cs_0.1_PbI_3_ perovskite: 4.0 μL of the precursor solution was supplied to the porous electrodes by drop-casting in a dry-air-filled glove box. The filled devices were kept with a petri dish cover at room temperature for 5 min, and then transferred to a 70 °C hot plate with a petri dish cover and kept for 2 h.

### 2.3. Encapsulation

Thermoplastic ionomer films were used as the encapsulant. The films were placed between the cells and cover glass. The difference between the over- and side-sealing methods is as follows: In over-sealing, the encapsulant covers the entire edge of the cell and the top of the cell. In side-sealing, the encapsulant seals only the edge of the cell. The samples were laminated at 110 °C for 5 min using a commercial vacuum laminator (PVL0202S, Nisshinbo Mechatronics, Tokyo, Japan) [20].

### 2.4. Characterization

The surface of the carbon electrode with perovskite crystals and the cross-section of the MPLE-PSC devices were observed using a scanning electron microscope (SEM, JSM-6510, JEOL Ltd., Tokyo, Japan). The photocurrent density–voltage (J-V) curves were measured with a DC voltage current source (B2901A, Agilent, Santa Clara, CA, USA) under a solar simulator (AM1.5G, 100 mW cm^−2^) equipped with a 500 W xenon lamp (YSS-100A, Yamashita Denso, Tokyo, Japan). The irradiation intensity of the AM1.5G solar simulator was calibrated using a reference silicon photodiode (Bunkoukeiki Co., Ltd., Tokyo, Japan). The light-irradiation area was 0.3 × 0.3 cm (0.09 cm^2^). The measurement voltage ranged from −0.05 to 1.05 V with forward and reverse scans, the step was set to 0.01 V, the integration time was set to 16.7 ms, and the scan delay time was 10 ms. The incident photon-to-current efficiency (IPCE) spectra were measured using a 150 W Xe lamp (TSM-K1, BSO-X150) equipped with a monochromator (MHM-K1) as a monochromatic light source. Calibration with the silicon photodiode (Bunkoukeiki Co., Ltd., Japan) was carried out before the IPCE measurements. The IPCE measurement was performed three times, and the interval was set to 3 min for the weak photoactivation. For more information on IPCE measurement methods for MPLE-PSCs, please refer to previous papers by the authors [36]. For J-V and IPCE measurements, the stabilized results of the 3rd measurement were used and compared. Electrochemical impedance spectroscopy (EIS) of the devices was performed on electrochemical workstations (Bio-Logic, Seyssinet-Pariset, France) in the frequency range from 1 Hz to 100 kHz at 0 V bias under the light. The voltage amplitude was 20 mV. The thermal stability of the perovskite crystals was characterized by X-ray diffraction (XRD, MiniFlex II, Rigaku, Tokyo, Japan). CuKα (λ = 1.5418Å) was used as the X-ray target. The scanning mode was continuous from 10 to 18°, with a sampling width of 0.01° and a scanning speed of 1 °/min.

### 2.5. Stability Test

The thermal stability test was performed by keeping the device on a hot plate at 85 °C in an ambient atmosphere (RT, 20–60%RH). During measurement and analysis, the devices were taken down from the hot plate and returned to room temperature before measurement and analysis were performed.

## 3. Results and Discussion

The compositions of the perovskite crystals used for the MPLE-PSCs in this study were (5-AVA)_0.05_(MA)_0.95_PbI_3_ and FA_0.9_Cs_0.1_PbI_3_. Herein, these perovskite compositions are simplified and referred to as MA perovskite and FACs perovskite, respectively.

### 3.1. Morphology of PSCs Used in Thermal Stability Test

The MA and FACs perovskites compared in this study had different perovskite crystal states due to the presence of additives and different crystallization conditions. Perovskite crystals were contained within m-TiO_2_, m-ZrO_2_, and mesoporous carbon layers with a total thickness of ~20 μm. Figure S1a,b show the SEM images of the perovskite crystals deposited on the carbon electrode surface of the MPLE-PSCs with MA and FACs perovskites. The crystal grain size showed an obvious difference between MA and FACs perovskites, with MA perovskite having a grain size of about 20 μm, while FACs perovskite had larger grains at about 500 μm. The reason for the smaller MA perovskite grains may be due to the addition of the 5-AVAI as an additive. A. Mei et al. reported that the 5-AVA cations have a large effective ion radius exceeding the tolerance factor to replace the MA site, thus 5-AVA could grow on the surfaces of MAPbI_3_ grains and adjust the grain size of MAPbI_3_ [23]. For this reason, the grain size of MAPbI_3_ with 5-AVAI may be limited below a certain size. They have also reported that the addition of 5-AVAI to MAPbI_3_ increases the stability of the perovskite crystals by strengthening the grain boundaries and forming strong bonds with TiO_2_ and ZrO_2_ nanoparticles [23]. On the other hand, it is believed that the larger crystal grain size observed in FACs perovskite can be attributed to the use of the solvent evaporation controlled crystallization (SECC) method, as proposed by Q. Wang et al. [31]. This method allows for a relatively slower crystallization process to occur, which may contribute to the formation of larger crystal grains. These grain boundaries can be the starting point for degradation. The 25-fold difference in crystal size between the two perovskite absorbers is due to the difference in the method used to densely pack the perovskite crystals within the multiporous layered structure. In MA perovskite, this is due to the 5-AVAI cation’s effect on grain size, while in FACs perovskite, it is due to the SECC method, which allows slow crystal growth without the use of additive molecules. The SECC method is based on the Ostwald ripening phenomenon, in which small particles shrink and disappear and larger particles grow in a system of particles of different sizes dispersed in the matrix phase [31]. This allows the formation of large perovskite crystals. In addition, in FACs perovskite, grain boundaries between grains are large and prominent (Figure S1b).

The presence of multiple voids in both MPLE-PSCs was confirmed by cross-sectional SEM images (Figure S1c,d, the areas indicated by arrows). Thus, both MPLE-PSCs with MA and FACs perovskites had incomplete fillings of the perovskite crystals in the MPLE. These voids, as defects, can degrade device performance. The formation of these defects can be effectively suppressed by controlling the perovskite composition and the crystal growth process [31,42].

### 3.2. Initial Device Performance

**Figure 2:**
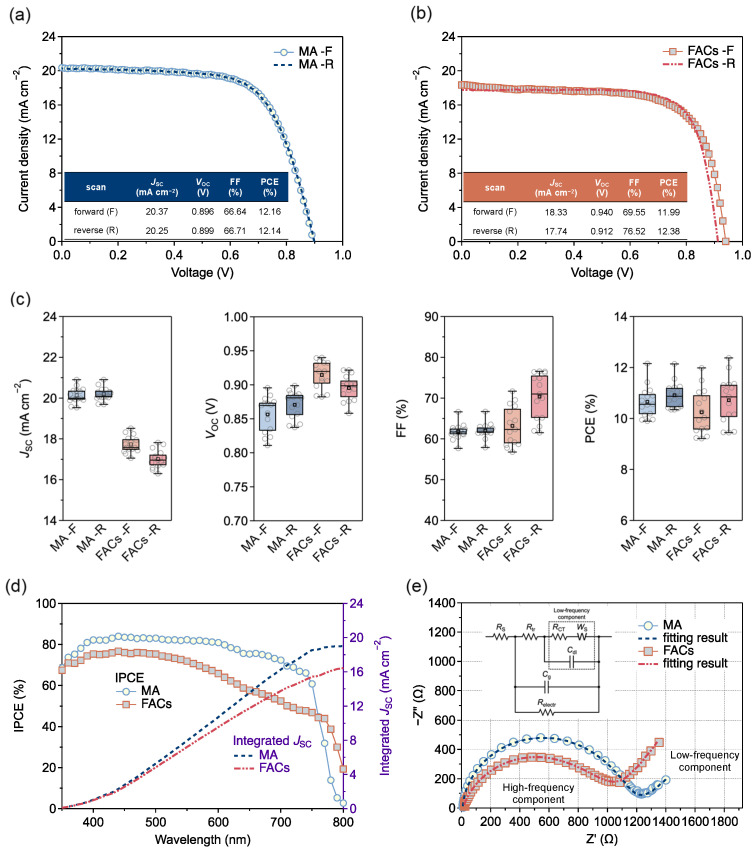
Device performance of MPLE-PSCs with MA and FACs perovskite. (**a**) J-V curves of MA perovskite. (**b**) J-V curves of FACs perovskite. (**c**) Box-and-whisker plots of each J-V parameter. (**d**) IPCE spectrum and integrated *J*_SC_. (**e**) Nyquist plots (the inset shows the equivalent circuit) of EIS at 0 V bias under the light.

The J-V, IPCE, and EIS results were carried out to compare and analyze the performances of the MPLE-PSCs with MA and FACs perovskites for the thermal stability tests. Figure 2a,b show the J-V curves for forward and reverse scans for the MPLE-PSCs with MA and FACs perovskites, respectively. Tables S1 and S2 show detailed device parameters. The parameters for the reverse scan for the champion devices were MA and FACs perovskites with a short circuit current density (*J*_SC_) of 20.25 mA cm^−2^ and 17.74 mA cm^−2^, open circuit voltage (*V*_OC_) of 0.899 V and 0.912 V, fill factor (FF) of 66.71% and 76.52%, and PCE of 12.14% and 12.38%, respectively. Figure 2c shows the box-and-whisker plots of the J-V parameters in 15 cells. The *J*_SC_ tended to be higher for MA perovskite than for FACs perovskite. On the contrary, the *V*_OC_ and FF tended to be higher for FACs perovskite than for MA perovskite. However, both devices had similar average PCE values of 10–11%.

From the IPCE spectra, FACs perovskite showed lower efficiencies than MA perovskite over the entire wavelength range (Figure 2d). The integrated *J*_SC_ was 19.02 mA cm^−2^ and 16.46 mA cm^−2^ for MA and FACs perovskite, respectively. The lower *J*_SC_ value for FACs perovskite can be attributed to the reduced active area due to grain boundaries, as seen in the SEM image. As a supplement, the slight difference between integrated *J*_SC_ and J-V *J*_SC_ is due to the leakage of scattered light from the glass substrate to the outside of the device without absorption by the photoactive area. This is due to the different optical diffusion (or parallel) characteristics of the two measurements [36].

In addition, the resistance component of the devices with MA and FACs perovskites was analyzed by EIS at 0 V bias under the light. The Nyquist plots are shown in Figure 2e and the resistivity parameters obtained from the fitting results are shown in Table S3. The EIS analysis was performed using the equivalent circuit model (Figure 2e), which was modeled as a mixed conductor system proposed by M. Bag et al. [43]. The model circuit consists of the following elements: The high-frequency component consists of the charge and ion transport resistance (*R*_tr_) coupled with the interfacial charge transfer resistance (*R*_CT_). The ion accumulation at the interface and the charge stored in the bulk perovskite layer are modeled as the interfacial Debye-layer capacitance (*C*_dl_) and the chemical capacitance (*C*_g_), respectively. Free carrier recombination/transport (both electrons and holes) is modeled by another resistance term (*R*_electr_). The series resistance (*R*_S_) represents the electrical resistance present on the carrier transport path in the device. The linear region in the low-frequency range is modeled by the Warburg diffusion (*W*_S_).

The *R*_S_ and *R*_electr_ were smaller in MA perovskite than in FACs perovskite. On the other hand, the *R*_tr_ and *R*_CT_ were smaller in FACs perovskite than in MA perovskite. Therefore, MA perovskite has a higher charge/ion transport resistance and interfacial charge transfer resistance, and a lower free carrier recombination/transport resistance than FACs perovskite. This can be attributed to the addition of 5-AVAI to MAPbI_3_, which limited the size of the perovskite crystals and increased defects [23]. Despite the differences in the device parameters, the PCEs of the MPLE-PSCs using MA and FACs perovskites were found to be almost similar. As a result, the MPLE-PSCs with similar PCEs worked for the comparisons of the thermal stability tests.

### 3.3. Thermal Stability of Unencapsulated Devices

**Figure 3:**
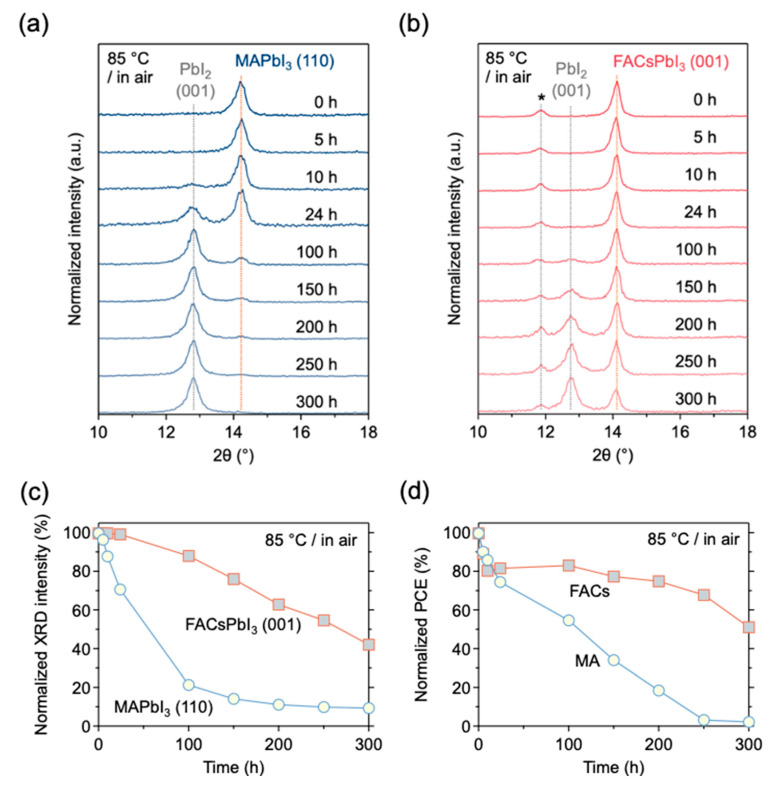
Thermal stability of MPLE-PSCs without any encapsulation at 85 °C in air. (**a**) Variation of XRD patterns of MAPbI_3_. (**b**) XRD pattern variation of FACsPbI_3_ (the reflections indicated by * represent Bragg reflections associated FAI). (**c**) Variation of normalized XRD intensities of MAPbI3 (110) and FACsPbI_3_ (001) perovskite crystals. (**d**) Variation of normalized PCE of MPLE-PSCs with MA and FACs perovskite.

The thermal stability at 85 °C of MPLE-PSCs with and without encapsulation was then investigated. First, the variation of properties with heat on unencapsulated devices was compared by XRD and J-V analysis. The results confirmed that, as is well known, MA perovskite crystals are more sensitive to the thermal environment than FACs perovskite crystals [44,45]. Figure 3a,b show the XRD patterns of MPLE-PSCs with MA and FACs perovskites in the 10–18° range. Each peak near 14° shows MAPbI_3_ (110) and FACsPbI_3_ (001). The peaks near 13° are attributed to PbI_2_ (001), and the peaks near 12° seen in FACs perovskite are associated with Bragg reflections of FAI [46]. The XRD peaks showing each perovskite crystal decreased with aging time, and the PbI_2_ peaks appeared simultaneously. The aging variation in these MA and FACs perovskites was compared (Figure 3c). The XRD intensity of MAPbI_3_ (110) decreased rapidly to about 20% of its initial value after 100 h. On the other hand, the XRD intensity of FACsPbI_3_ (001) remained at 90% after 100 h and decreased to 40% after 300 h. In addition, Figure 3d shows the aging variation of the PCE, which continued to be acquired simultaneously with the XRD measurements during the thermal stability test. The PCE of MPLE-PSC with MA perovskite crystals continued to decrease with aging time and reached almost 0% after 300 h. On the other hand, the PCE of MPLE-PSC with FACs perovskite decreased to 80% of its initial value in the first 10 h and maintained 50% of its initial value after 300 h. The initial degradation in the thermal stability test can be attributed to changes in the state of the perovskite crystals due to the transition from the device fabrication environment in a vapor solvent atmosphere to the thermal stability test in a dry atmosphere. Therefore, without encapsulation, both perovskites were degraded by heat, but it was more pronounced for the MA perovskite.

### 3.4. Thermal Stability of Encapsulated Devices

Surprisingly, the trend of stability was reversed in the encapsulated device, with MA perovskite showing higher thermal stability than FACs perovskite. Figure 1, Figures S2 and S3 show the schematic diagram, sealing procedure, and photographs of the over- and side-sealing methods. In the over-sealing structure, the top of the cell contacts the encapsulant, which covers the entire cell. In contrast, for a side-sealing structure, the top of the cell does not contact the encapsulant, which covers only the surrounding area of the cell. The difference between the two encapsulation structures is the presence or absence of space above the cell.

**Figure 4:**
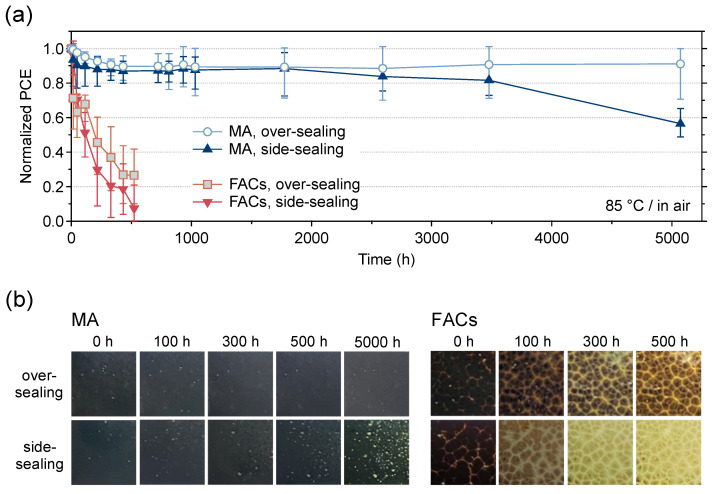
(**a**) Variation for normalized PCE at the initial value of encapsulated MPLE-PSCs during the thermal stability test (85 °C (ISOS-D-2) for >5000 h). The number of used devices was five for the statistical data. (**b**) Photographs of the active area during the thermal test as seen from the FTO glass side (light-receiving surface).

Thermal stability tests were performed by placing the MPLE-PSCs on a hot plate at 85 °C in an ambient atmosphere (Figure S4). Figure 4a shows the PCE variation from the initial value during thermal stability tests of encapsulated MPLE-PSCs with MA and FACs perovskites. In addition, Figures S5 and S6 show the variations of *J*_SC_, *V*_OC_, FF, *R*_s_, and *R*_sh_. In FACs perovskite, after 500 h, the PCE decreased to about 30% and 10% of the initial value for over- and side-sealing, respectively. These degradations were due to the overall degradation of the device performance, with the *J*_SC_ degradation rate being particularly significant. On the other hand, without encapsulation, it decreased to about 50% of the initial value after 300 h. Therefore, the FACs perovskite did not show any improvement in thermal stability after the encapsulation. It is known that the amine groups in FA can react with carbon via a condensation reaction, which may have accelerated the degradation of the perovskite crystals [47]. In addition, large grain boundaries exist between the large crystals of FACs perovskite. These grain boundaries are the area where the perovskite crystals are not packed. H_2_O in the encapsulated device could easily reach the perovskite crystals in the ZrO_2_ and TiO_2_ layers, and degradation could have proceeded immediately from these starting points.

On the other hand, the MA perovskite maintained about 90% of its initial PCE up to 1800 h for both encapsulation methods. The side-sealing devices showed a gradual decrease in the PCE, down to about 60% of the initial value after more than 5000 h. However, the over-sealing device surprisingly maintained 90% of its initial PCE over more than 5000 h. The lifetime of each device was then compared when its performance dropped to 80% of its initial value (*T*_80_ lifetime). The *T*_80_ lifetimes were 16, >5000, and 3580 h for without encapsulation, over-, and side-sealing for MA perovskite, and 125, 11, and 35 h for without encapsulation, over-, and side-sealing for FACs perovskite, respectively (Table S4). Initial degradation was observed for MA perovskite. This is explained by the burn-in effects observed in the early stages of the thermal stability test [35]. The ability to achieve high stability despite several defects in the perovskite crystals of the device, as seen in the cross-sectional SEM, indicates the high potential for commercialization of MPLE-PSCs. The reason for the improved thermal stability of MA perovskite can be attributed to the effective suppression of the MAPbI_3_ decomposition reaction by encapsulation.

The main degradation factors in these MA- and FACs-based MPLE-PSCs were the decrease in the *J*_SC_ and FF. Therefore, the changes in the IPCE and EIS during the thermal stability test were also investigated (Figures S7–S10). For FACs perovskite, the total IPCE continued to decrease and the resistive component continued to increase regardless of the encapsulation method. On the other hand, MA perovskite showed changes in the IPCE and EIS after 5000 h of the thermal stability test depending on the encapsulation method. No significant changes were observed in over-sealing, while side-sealing showed a decrease in the IPCE and an increase in the resistive component after 5000 h. Thus, the thermal degradation rate of perovskite varies depending on the perovskite crystal and the encapsulation method.

In addition, changes in the appearance of the active area of the device during the thermal stability test were compared (Figure 4b). In the MA perovskite, there was no noticeable change after 5000 h in the over-sealing devices. In the side-sealing device, several yellow dots appeared in the active area after 5000 h. This is attributed to the decomposition of the perovskite crystals into PbI_2_. Interestingly, the yellow dots associated with PbI_2_ precipitation did not appear at the edges of the cell, but rather throughout the active area. This can be attributed to the presence of space above the cell, even though the area around the cell was completely sealed by the encapsulant. On the other hand, for the FACs perovskite, the perovskite decomposed and turned yellow during the thermal test regardless of the encapsulation method. The degree of discoloration was more pronounced on the side-sealing device. Therefore, regardless of the perovskite composition, side-sealing would have a more negative effect on the device than over-sealing.

As supporting information, a similar thermal stability test was performed at 100 °C, which is 15 °C higher than the 85 °C specified in the ISOS-D-2 protocol, to confirm thermal stability at even higher temperatures (Figures S11 and S12). The difference in thermal stability between the perovskite composition and encapsulation method combinations was the same as for the 85 °C case. However, for all combinations, the thermal stability was lower than at 85 °C. The *T*_80_ lifetime was 432, 22, 48, and 15 h for over- and side-sealing for MA perovskite and for over- and side-sealing for FACs perovskite, respectively (Table S5). At 100 °C, the combination of MA perovskite and over-sealing showed the highest stability, with a change from the initial value of about 70% at 3500 h and about 40% at 5000 h. Thus, it should be noted that for MA-based perovskites, there is a temperature limit for which the cell can be protected by proper encapsulation.

### 3.5. Degradation Mechanisms for Over- and Side-Sealing Devices

Based on these results, we propose a thermal degradation model for the effect of over- and side-sealing using thermoplastic resin films.

**Figure 5:**
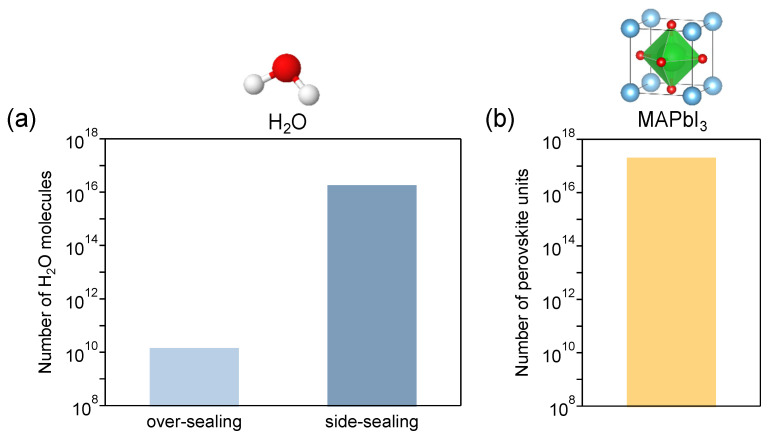
(**a**) Calculation of the number of H_2_O molecules sealed in the 1 cm^2^ active area device by over- and side-sealing, and (**b**) the number of perovskite units in the device.

As a prerequisite, all processes including the encapsulation of the MPLE-PSCs fabricated in this study were performed under an ambient atmosphere, which is not a special environment. Therefore, the encapsulated device contains a certain amount of water (H_2_O) depending on the encapsulation method. The amount of H_2_O molecules contained in the device with over- and side-sealing was estimated by calculation (the details of the calculation are described in Supplementary Text S1). The estimated numbers of H_2_O molecules sealed in the over-sealing and side-sealing devices are 1.47 × 10^10^ and 1.85 × 10^16^, respectively (Figure 5a). This amount of H_2_O in the side-sealing method is 1,263,160 times the amount in the over-sealing method. The large difference is due to the space formed by the side-sealing method. The H_2_O has a catalytic effect and reacts with MAPbI_3_ to decompose the perovskite crystals [48]. The decomposition reaction equation for MAPbI_3_ under the influence of water is shown below.

(1) $${\mathrm{CH}}_{3}{\mathrm{NH}}_{3}{\mathrm{PbI}}_{3}\;\overset{H_{2}O}{⇄}{\mathrm{CH}}_{3}{\mathrm{NH}}_{3}{\mathrm{PbI}}_{3}\cdot H_{2}O,{\left({\mathrm{CH}}_{3}{\mathrm{NH}}_{3}\right)}_{4}{\mathrm{PbI}}_{6}\cdot 2H_{2}O\;\to {\mathrm{CH}}_{3}{\mathrm{NH}}_{2}\;(\mathrm{gas})+{\mathrm{PbI}}_{2}+\mathrm{HI}\;(\mathrm{gas})+\mathrm{other}\;\mathrm{compositions}$$

Here, the number of perovskite units in the devices is estimated to be 2.04 × 10^12^ (Figure 5b, the details of the calculation are described in Supplementary Text S2). The number of perovskite crystal units is approximately 13,000,000 and 11 times the number of H_2_O molecules in the over- and side-sealing devices, respectively. Thus, the number of H_2_O molecules in the side-sealing device is sufficient to fracture the perovskite crystals.

Furthermore, it has been reported that in MPLE-PSCs, MAPbI_3_ perovskite crystals in the multiporous layered electrode decompose in a thermal environment. Z. Fu et al. showed that after approximately 200 h of thermal stability testing at 85 °C in an unencapsulated (5-AVA)_x_(MA)_1−x_PbI_3_-based MPLE-PSC device, holes formed in the TiO_2_/ZrO_2_ layer and the perovskite absorber decomposed [27]. In addition, A. K. Baranwal et al. disassembled side-sealing MAPbI_3_-based MPLE-PSC devices after 7000 h of thermal stability testing at 100 °C and analyzed the internal perovskite state [29]. They found that MA^+^ species in MA-based perovskites readily react with carbon under applied thermal stress, resulting in perovskite crystal decomposition.

E.J. Juarez-Perez et al. experimentally demonstrated that MAPbI_3_ can release NH_3_ and CH_3_I as decomposition products by thermal decomposition in the temperature range of 20–600 °C and under atmospheric pressure [49]. F. Fu et al. also reported a self-propagating degradation mechanism associated with I_2_ vapor release in FA-based PSCs stressed under operating conditions of 80 °C in a N_2_ atmosphere [50]. However, MPLE-PSCs with carbon electrodes may have a different degradation mechanism than typical thin-film PSCs.

Thus, in a thermal environment, two mechanisms are possible: water acts as a catalyst to decompose perovskite, and carbon reacts with MA^+^ species to decompose perovskite. Since the decomposition of perovskite is thought to occur through a combination of these factors, it is difficult to isolate these mechanisms.

**Figure 6:**
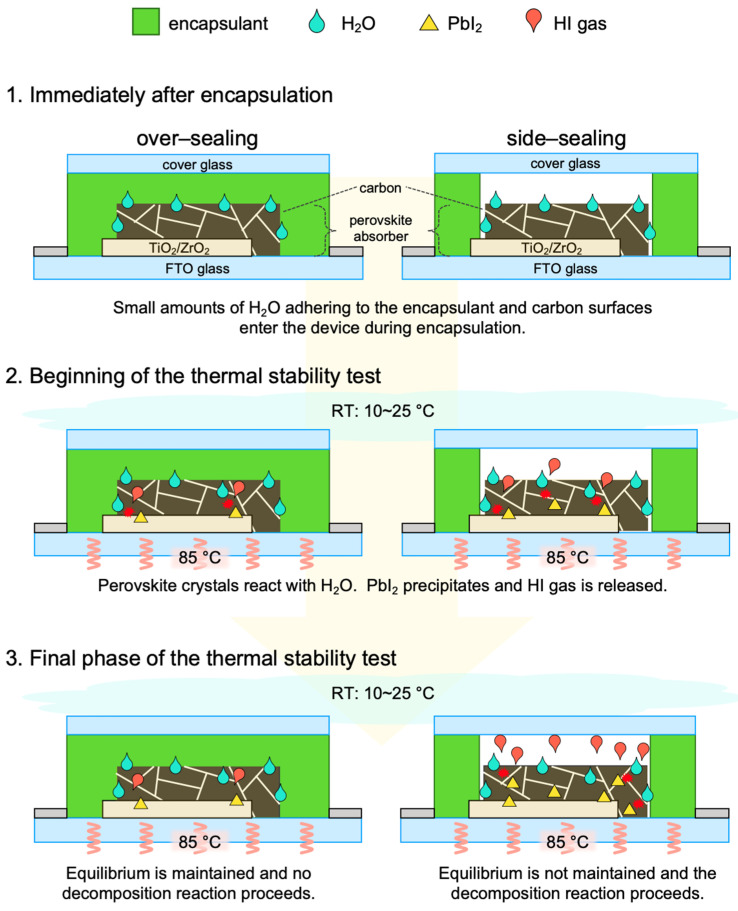
Thermal degradation model of MPLE-PSC with over- and side-sealing encapsulation.

Based on these results and assumptions, when encapsulated cells are placed in a thermal environment, the following phenomena are expected to occur, depending on the encapsulation method, as shown in Figure 6.

Over-sealing: 1. Immediately after encapsulation in an ambient atmosphere, the device is completely covered by the encapsulant and contains a small amount of H_2_O between the encapsulant and the device. The estimated number of H_2_O molecules sealed in the over-sealing device is 1.47 × 10^10^. 2. When the thermal stability test starts, the heated H_2_O becomes water vapor and permeates and enters the cell. The equilibrium reaction shown in Equation (1) proceeds, some perovskite crystals are decomposed, PbI_2_ is precipitated, and HI gas is released. 3. However, the amount of H_2_O sealed in the over-sealing device is relatively small. Therefore, the equilibrium condition in Equation (1) is expected to be maintained and the precipitation of PbI_2_ and the release of HI gas will not exceed a certain amount. Therefore, the degradation of perovskite crystals is not expected to progress beyond a certain level that has reacted at the beginning of the thermal stability test.

Side-sealing: 1. Immediately after encapsulation under an ambient atmosphere, an enclosed space is formed between the device and the cover glass, trapping air containing H_2_O. In addition, H_2_O adheres to the encapsulant and device. The estimated number of H_2_O molecules sealed in the side-sealing device is 1.85 × 10^16^. This amount of H_2_O is approximately 1,260,000 times the amount in the over-sealing device. 2. At the beginning of the thermal stability test, the perovskite crystals in the carbon layer react with H_2_O to precipitate PbI_2_ and release HI gas. This HI gas is released toward the space between the device and the encapsulating components. Therefore, the equilibrium reaction of the device, given by Equation (1), is no longer valid, and only the positive reactions will continue to proceed. In addition, the top of the cover glass is in contact with the surrounding atmosphere (RT: 10~25 °C), which constantly cools the space between the device and the cover glass. This may cool the H_2_O and volatile gases in the space and reduce the gas pressure in the space, which further promotes the HI gas volatilization of the device. 3. Once the equilibrium is lost, the perovskite crystals continue to decompose. In addition, humid air containing H_2_O from outside of the device can enter the device through a small gap between the encapsulant of the side-sealing and the adhesive surface of the cover glass. In the final phase of the thermal stability test, the decomposition reaction will eventually progress to the perovskite crystals in the exposed m-ZrO_2_ and m-TiO_2_ layers, and the device performance is expected to deteriorate as a result.

## 4. Conclusions

Solving the problems of cost and stability is the main key to the practical application of PSCs. In this study, the thermal stability (at 85 °C) of low-cost carbon-based MPLE-PSCs was investigated. The light absorbers (MA and FACs perovskites), as well as the encapsulation methods of over- and side-sealing with thermoplastic resin films, were compared. As a result, without encapsulation, FACs perovskite showed higher thermal stability than MA perovskite. However, with encapsulation, the phenomenon of thermal stability was reversed as the MA perovskite showed higher thermal stability than the FACs perovskite. It is believed that the thermal decomposition reaction of MAPbI_3_ was effectively suppressed by encapsulating the device. On the other hand, the thermal stability of MPLE-PSCs with FACs perovskites was not improved by encapsulation. This was an issue of crystal quality and the material of the FACs perovskite. In particular, the condensation reaction between the amine groups in the FACs and carbon can occur in the encapsulated cells, resulting in decomposition of the perovskite crystals starting from the grain boundaries.

Using a thermoplastic ionomer sealant, the over-sealing devices in this study showed higher thermal stability than the side-sealing devices. For MA perovskite, the normalized PCE at the initial value after >5000 h was approximately 90% and 60% for over- and side-sealing devices, respectively. It was estimated that approximately 1,260,000 times more H_2_O is trapped in the cell in side-sealing devices than in over-sealing devices due to the space at the top of the cell. Because of this relatively large amount of H_2_O, the side-sealing device degraded more quickly. In addition, because the upper part of the space is always cooled by cold air from the outside, the gas pressure in the space decreases and equilibrium reactions are not established. Once equilibrium was lost, the decomposition of the perovskite crystals continued to progress. On the other hand, in the over-sealing device, the top of the cell is completely covered by the encapsulant, which suppresses the release of these volatiles to the outside of the cell. These results indicate that over-sealing encapsulation can significantly extend the thermal stability of devices, even when using MA perovskites with low thermal stability. However, it should be noted that different encapsulant materials may change the optimal encapsulation method. Therefore, this study demonstrates the high reliability of commercialization for carbon-based MPLE-PSCs with a thermoplastic ionomer sealant.

## Data Availability

The original contributions presented in the study are included in the article/Supplementary Material, further inquiries can be directed to the corresponding authors.

## Supplementary Materials

The following supporting information can be downloaded at: https://www.mdpi.com/article/10.3390/ma17123002/s1, Text S1: Calculation of the amount of H_2_O sealed in the encapsulated device; Text S2: Calculation of the number of perovskite units in the devices; Figure S1: SEM images of the surface and the cross-section of MPLE-PSCs using MA and FACs perovskites; Table S1: Device parameters of MA-based MPLE-PSCs; Table S2: Device parameters of FACs-based MPLE-PSCs; Table S3: Resistance parameters obtained from EIS measurement at 0 V bias under the right and fitting; Figure S2: The sealing procedure of MPLE-PSCs; Figure S3: Photographs of encapsulated MPLE-PSCs; Figure S4: Encapsulated MPLE-PSCs during thermal stability test; Figure S5: Variations for normalized *J*_SC_, *V*_OC_, and FF at the initial value of encapsulated MPLE-PSCs at thermal stability test; Figure S6: Variations for normalized *R*_s_ and *R*_sh_ at the initial value of encapsulated MPLE-PSCs at thermal stability test; Table S4: The time when each device’s performance has degraded to 80% of its initial value (*T*_80_ lifetime) in a thermal stability test at 85 °C; Figure S7: Changes in IPCE spectrum and integrated *J*_SC_ of MPLE-PSCs with FACs perovskite during thermal stability tests; Figure S8: Changes in Nyquist plots of MPLE-PSCs with FACs perovskite during thermal stability tests; Figure S9: Changes in IPCE spectrum and integrated *J*_SC_ of MPLE-PSCs with MA perovskite during thermal stability tests; Figure S10: Changes in Nyquist plots of MPLE-PSCs with MA perovskite during thermal stability tests; Figure S11: Variations for normalized PCE, *J*_SC_, *V*_OC_, and FF at the initial value of encapsulated MPLE-PSCs at thermal stability test (100 °C for >5000 h); Figure S12: Variations for normalized *R*_s_ and *R*_sh_ at the initial value of encapsulated MPLE-PSCs at thermal stability test (100 °C for >5000 h); Table S5: The time when each device’s performance has degraded to 80% of its initial value (*T*_80_ lifetime) in a thermal stability test at 100 °C. Reference [51] is cited in the Supplementary Materials.
