# Supplementary material for: Thermal Stability of Encapsulated Carbon-Based Multiporous-Layered-Electrode Perovskite Solar Cells Extended to Over 5000 h at 85 °C
Source: Materials (Basel). 2024 Jun 19;17(12):3002. doi: 10.3390/ma17123002 (PMC11205374; doi:10.3390/ma17123002)
Supplement: Supplementary file 1 [file materials-17-03002-s001.zip › materials-3043460-supplementary.pdf]

Supplementary Materials

Article

Thermal Stability of Encapsulated Carbon-Based Multiporous-Layered-Electrode Perovskite Solar Cells Extended to Over 5000 h at 85 °C

Ryuki Tsuji[1,2*], Yuuma Nagano[1], Kouta Oishi[1], Eiji Kobayashi[2], and Seigo Ito[1*]

[1] Department of Materials and Synchrotron Radiation Engineering, Graduate School of Engineering, University of Hyogo, 2167 Shosha, Himeji 671-2280, Hyogo, Japan

[2] Department of Materials Science, Institute of Pure and Applied Sciences, University of Tsukuba, 1-1-1 Tennodai, Tsukuba 305-8573, Ibaraki, Japan

[3] Kishu Giken Kogyo Co., Ltd., 446 Nunohiki, Wakayama 641-0015, Wakayama, Japan

* Correspondence: tsuji.ryuki.gp@u.tsukuba.ac.jp (R.T.); itou@eng.u-hyogo.ac.jp (S.I.); Tel.: +81-79-267-4150 (S.I.)

Contents:

Supplementary text S1-S2, Figure S1-S12, Table S1-S5

Supplementary text

S1. Calculation of the amount of H_2O sealed in the encapsulated device

Assume that a single molecular layer of H_2O (molecular size: 0.38 nm) exists adsorbed on the cell and encapsulation components surfaces. Therefore, to calculate the amount of H_2O molecules present on the cell and encapsulant surfaces, the height of a single layer of H_2O molecules was taken into account for the volume calculation. In side-sealing, H_2O molecules are adsorbed on the cell surface, encapsulant surface, and cover glass surface, and their volumes are 0.038 mm^3 (length: 100 mm × width: 100 mm × height: 0.38 nm), 0.000076 mm^3 (length: 100 mm × width: 0.5 mm × height: 3.8 nm × 4 sides), and 0.038 mm^3 (length: 100 mm × width: 100 mm × height: 0.38 nm), respectively. Additionally, the volume of the space between the cell and the encapsulation components is 4800 mm^3 {length: 100 mm × width: 100 mm × height: 0.48 mm (Height of encapsulant: 0.5 mm - height of the cell: 0.02 mm = 0.48 mm)}. The following is a calculation of the estimated amount of sealed H_2O molecules (*est.* H_2O amount) based on these total volumes and the amount of water vapor at room temperature (25 °C, 23.1 g m^{-3}). Relative humidity was set at 50%.

est. H_2O amount in side-sealing device:

$$23.1 \text{ g m}^{-3} / 0.50 \times (0.038 + 0.000076 + 0.038 + 4800 \text{ mm}^3) = 5.44\times10^{-5} \text{ g}$$

Converting to molecule amount: $(5.44\times10^{-5} \text{ g} / 18.02 \text{ g mol}^{-1}) \times 6.02\times10^{23} \text{ mol}^{-1} = 1.85\times10^{16}$

(Molar volume of H_2O: 18.02 g/mol, Avogadro constant: 6.02×10^{23} mol^{-1})

In over-sealing, H_2O on the surface of the cell and encapsulant is sealed into the device after encapsulation. Assuming this to be a monolayer, the volume is 0.038 mm^3 (length: 100 mm × width: 100 mm × height: 0.38 nm). As with side-sealing, from these total volumes, the estimated amount of H_2O molecules sealed in the over-sealing device can be calculated as follows.

est. H_2O amount in over-sealing device: $23.1 \text{ g m}^{-3} / 0.50 \times 0.038 \text{ mm}^3 = 4.39\times10^{-11} \text{ g}$

Converting to molecule amount: $(4.39\times10^{-11} \text{ g} / 18.02 \text{ g mol}^{-1}) \times 6.02\times10^{23} \text{ mol}^{-1} = 1.47\times10^{10}$

S2. Calculation of the number of perovskite units in the devices

The number of $MAPbI_3$ perovskite crystal units was calculated assuming that the perovskite crystals are packed into the m-TiO_2 and m-ZrO_2 layers (2 μm) of a 1 cm^2 device. The volume of perovskite crystals contained in a 1 cm^2 device is:

$$10\ mm \times 10\ mm \times 0.002\ mm = 0.2\ mm^3$$

The volume of one unit of $MAPbI_3$ perovskite was 0.98 nm^3.[51] For simplicity, porous structures are not considered here. The number of perovskite crystal units is as follows.

$$0.2\ mm^3 / 9.8 \times 10^{-19}\ mm^3 = 2.04 \times 10^{17}$$

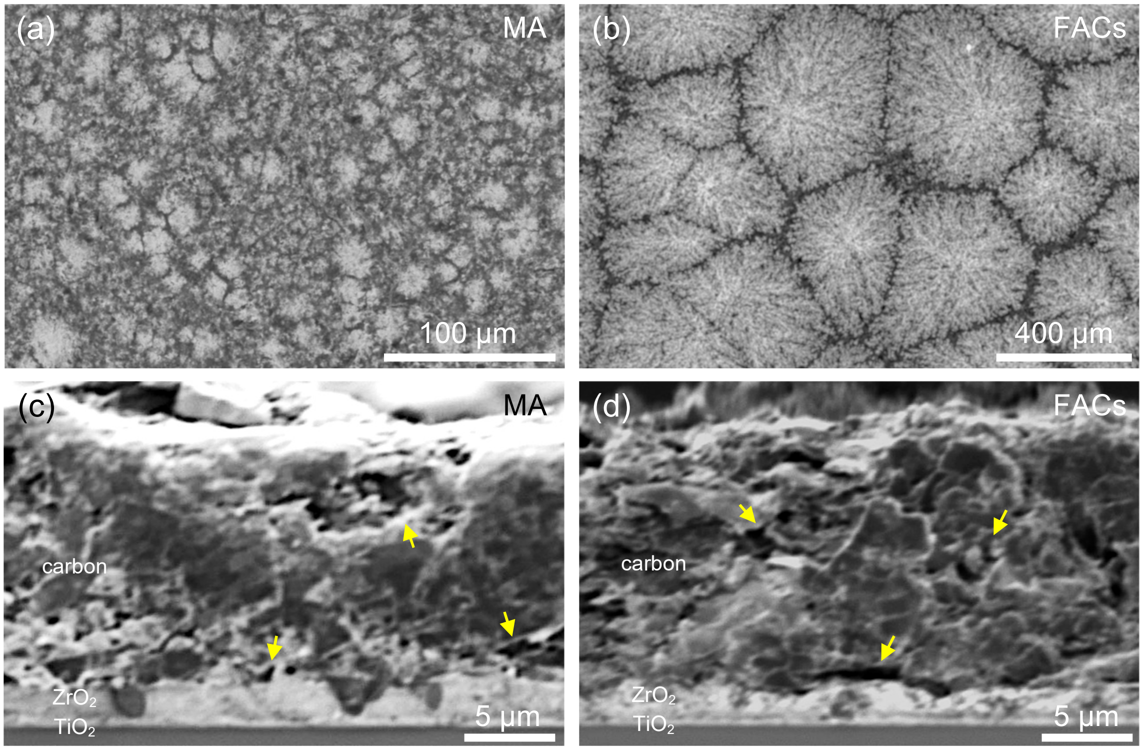

Figure S1. SEM images of the surface (a, b) and the cross-section (c, d) of MPLE-PSCs using MA (a, c) and FACs (c, d) perovskites.

Table S1. Device parameters of MA-based MPLE-PSCs.

	PCE, rev. (%)	PCE, fwd. (%)	J_{SC}, rev. (mA cm^{-2})	J_{SC}, fwd. (mA cm^{-2})	V_{OC}, rev. (V)	V_{OC}, fwd. (V)	FF, rev. (%)	FF, fwd. (%)
**cell 1**	10.43	9.96	20.08	19.99	0.837	0.823	62.01	60.52
**cell 2**	10.97	10.71	20.41	20.36	0.870	0.858	61.71	61.28
**cell 3**	11.18	11.01	20.04	19.94	0.886	0.872	62.98	63.33
**cell 4**	10.70	10.54	19.70	19.53	0.884	0.870	61.48	62.05
**cell 5**	11.06	10.86	20.06	20.00	0.881	0.871	62.57	62.35
**cell 6**	10.48	10.19	20.29	20.33	0.840	0.821	61.51	61.09
**cell 7**	11.56	11.43	20.91	20.90	0.888	0.880	62.30	62.14
**cell 8**	11.22	11.11	20.67	20.63	0.881	0.873	61.59	61.71
**cell 9**	10.35	10.15	20.04	19.89	0.892	0.885	57.90	57.69
**cell 10**	10.75	10.56	20.31	20.19	0.882	0.875	59.99	59.78
**cell 11**	11.25	10.90	20.42	20.43	0.867	0.849	63.56	62.81
**cell 12**	10.87	10.46	20.08	19.98	0.858	0.835	63.14	62.69
**cell 13**	10.40	9.90	20.01	19.92	0.842	0.811	61.68	61.28
**cell 14**	10.35	9.95	19.70	19.58	0.854	0.830	61.51	61.20
**cell 15**	12.14	12.16	20.25	20.37	0.899	0.896	66.71	66.64
**Max value**	12.14	12.16	20.91	20.90	0.899	0.896	66.71	66.64
**Average**	10.91	10.66	20.20	20.14	0.871	0.856	62.04	61.77
**Standard deviation**	±0.49	±0.6	±0.31	±0.36	±0.019	±0.026	±1.81	±1.86

Table S2. Device parameters of FACs-based MPLE-PSCs.

	PCE, rev. (%)	PCE, fwd. (%)	J_{SC}, rev. (mA cm^{-2})	J_{SC}, fwd. (mA cm^{-2})	V_{OC}, rev. (V)	V_{OC}, fwd. (V)	FF, rev. (%)	FF, fwd. (%)
**cell 1**	10.60	10.03	16.49	17.44	0.918	0.932	70.00	61.72
**cell 2**	10.16	9.63	16.30	17.06	0.900	0.905	69.24	62.33
**cell 3**	9.46	9.22	17.08	17.83	0.894	0.884	61.97	58.50
**cell 4**	12.38	11.99	17.74	18.33	0.912	0.940	76.52	69.55
**cell 5**	11.36	11.03	16.85	17.39	0.901	0.932	74.88	68.04
**cell 6**	11.21	11.08	17.14	17.50	0.858	0.882	76.18	71.71
**cell 7**	11.08	10.60	16.69	17.27	0.887	0.920	74.87	66.73
**cell 8**	10.16	9.94	17.82	18.52	0.876	0.898	65.13	59.72
**cell 9**	10.85	10.27	16.96	17.58	0.874	0.906	73.23	64.52
**cell 10**	12.00	11.50	17.57	18.02	0.892	0.929	76.57	68.65
**cell 11**	9.45	9.30	17.06	17.74	0.900	0.885	61.53	59.18
**cell 12**	11.33	10.79	17.31	17.95	0.922	0.940	71.01	64.01
**cell 13**	9.48	9.24	16.73	17.57	0.899	0.909	63.04	57.80
**cell 14**	9.96	9.61	16.58	17.50	0.922	0.935	65.19	58.69
**cell 15**	11.31	9.53	16.87	18.25	0.877	0.920	76.40	56.79
**Max value**	12.38	11.99	17.82	18.52	0.922	0.940	76.57	71.71
**Average**	10.72	10.25	17.01	17.73	0.895	0.915	70.39	63.20
**Standard deviation**	±0.89	±0.85	±0.43	±0.4	±0.018	±0.02	±5.5	±4.65

Table S3. Resistance parameters obtained from EIS measurement at 0 V bias under the right and fitting.

light absorber	R_s (Ω)	R_{tr} (Ω)	C_g (F)	R_{CT}	C_{dl} (F)	W_s (Ω)	R_{electr} (Ω)
MA	15.6	1097	5.5×10^{-11}	31.0	1.2×10^{-7}	509	29221
FACs	23.7	861.8	4.9×10^{-12}	21.3	2.1×10^{-7}	586	38420

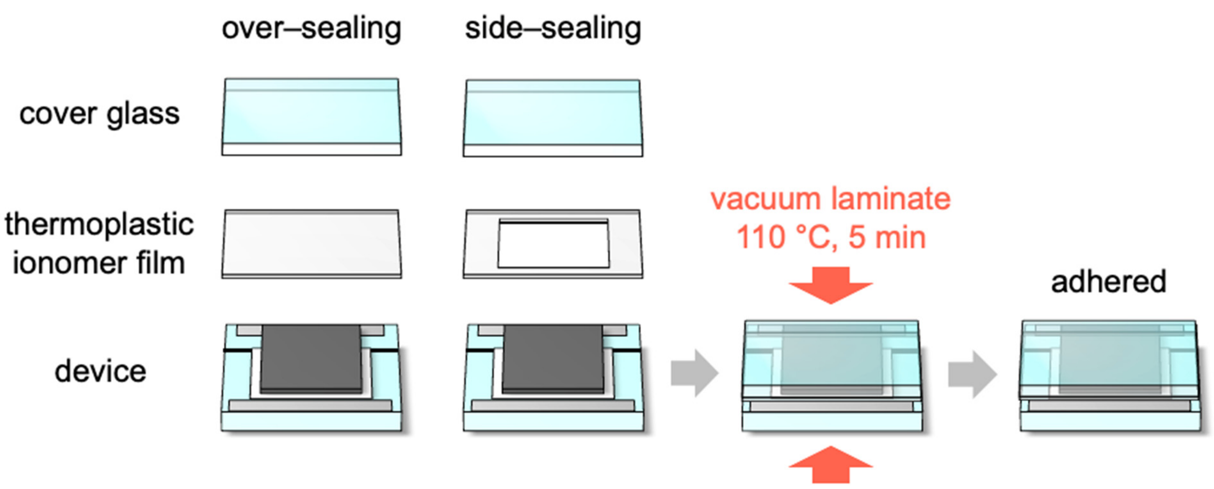

Figure S2. The sealing procedure of MPLE-PSCs.

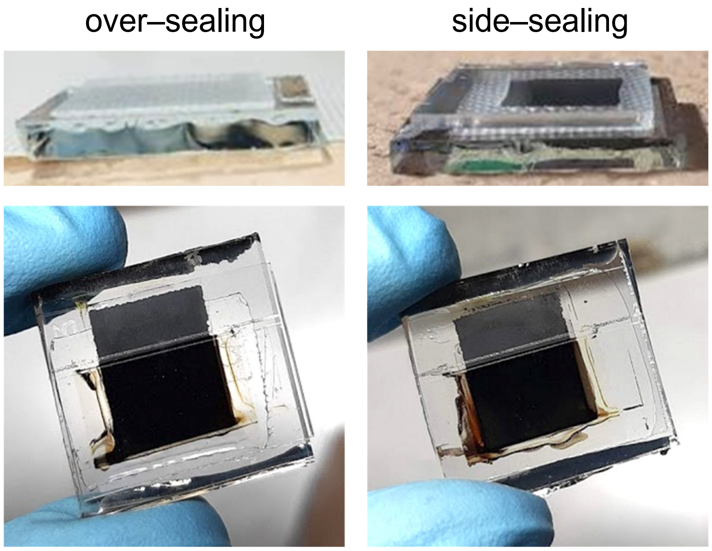

Figure S3. Photographs of encapsulated MPLE-PSCs.

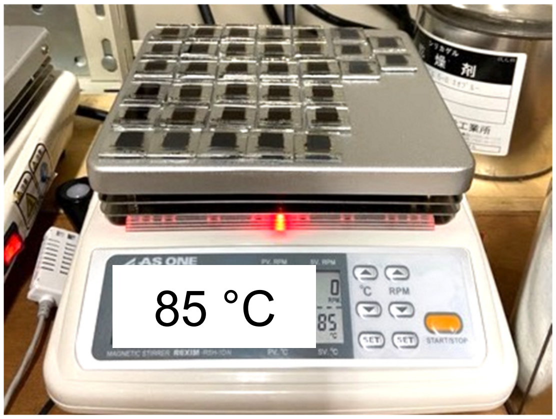

Figure S4. Encapsulated MPLE-PSCs during thermal stability test.

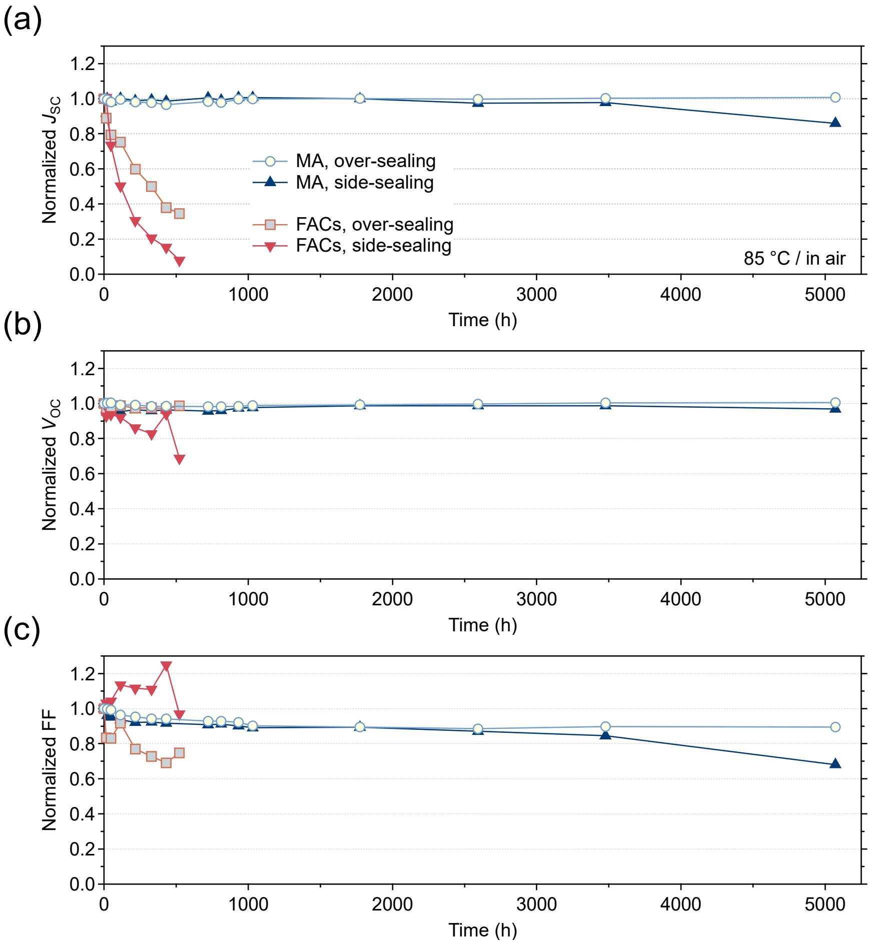

Figure S5. Variations for normalized (a) J_{SC}, (b) V_{OC}, and (c) FF at the initial value of encapsulated MPLE-PSCs at thermal stability test (85 °C (ISOS-D-2) for >5,000 h). The number of used devices was five for the statistical data, and only the average values were plotted.

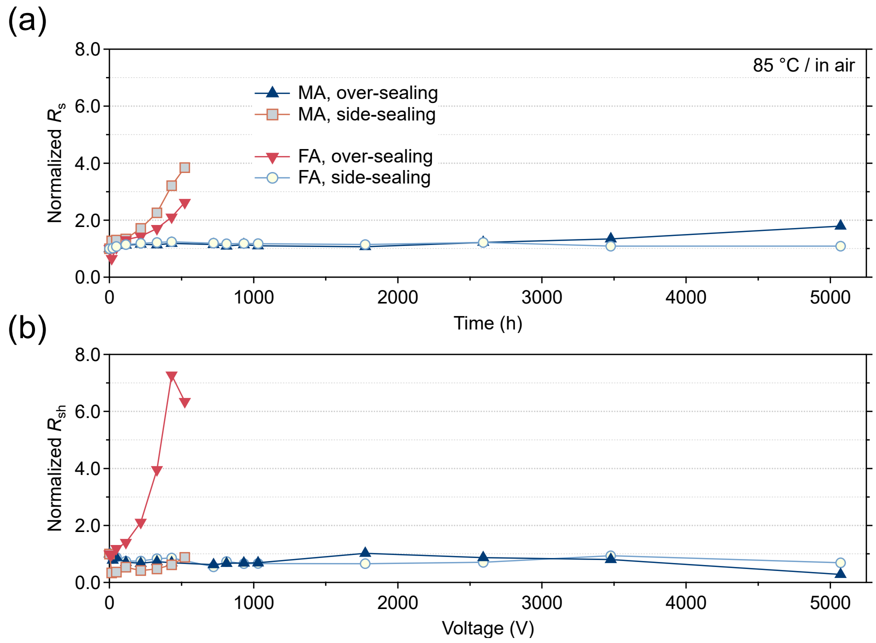

Figure S6. Variations for normalized (a) R_s and (b) R_{sh} at the initial value of encapsulated MPLE-PSCs at thermal stability test (85 °C (ISOS-D-2) for >5,000 h). The number of used devices was five for the statistical data, and only the average values were plotted.

Table S4. The time when each device's performance has degraded to 80% of its initial value (T_{80} lifetime) in a thermal stability test at 85 °C. The average times are shown. The number of used devices was five for the statistical data.

light absorber	w/o encapsulation	over-sealing	side-sealing
MA	16 h	>5000 h	3580 h
FACs	125 h	11 h	35 h

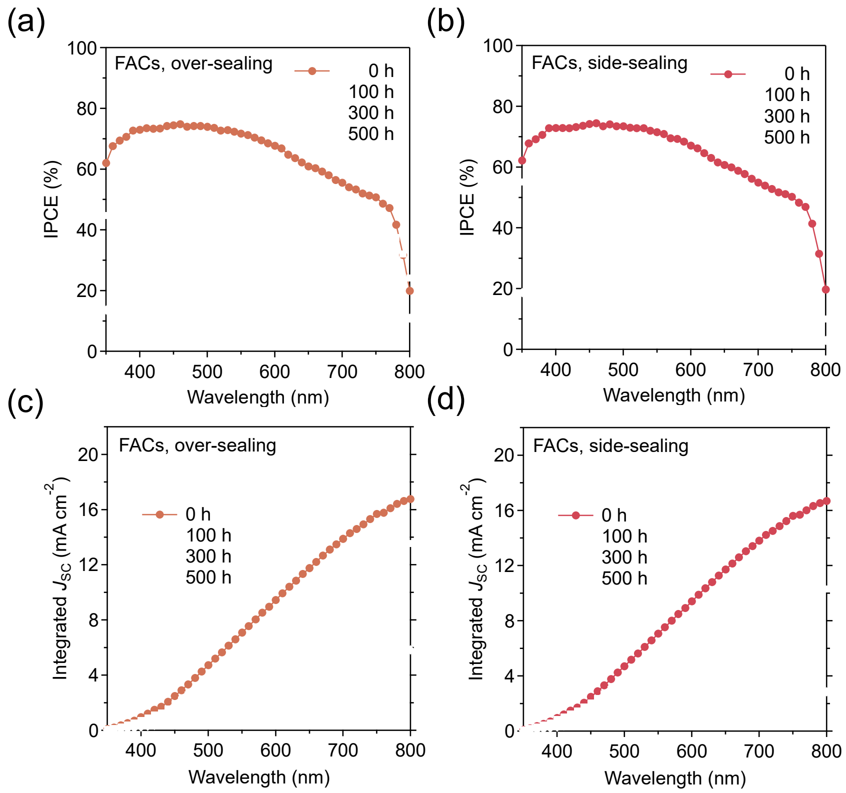

Figure S7. Changes in IPCE spectrum and integrated J_{SC} of MPLE-PSCs with FACs perovskite during thermal stability tests. (a, c) over-sealing and (b, d) side-sealing.

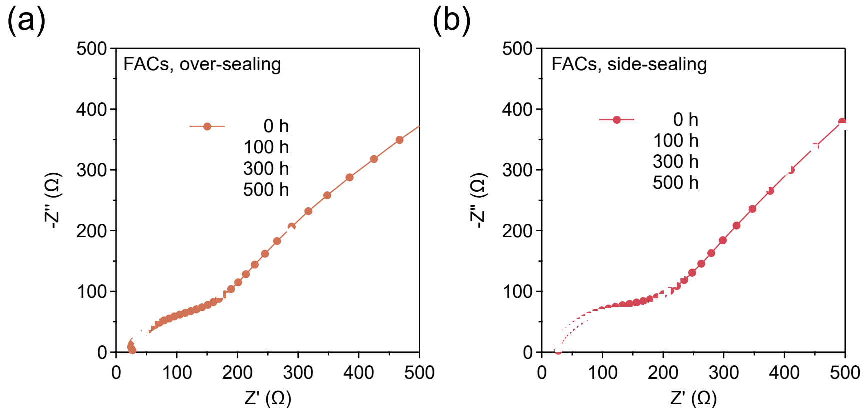

Figure S8. Changes in Nyquist plots of MPLE-PSCs with FACs perovskite during thermal stability tests. (a) over-sealing and (b) side-sealing.

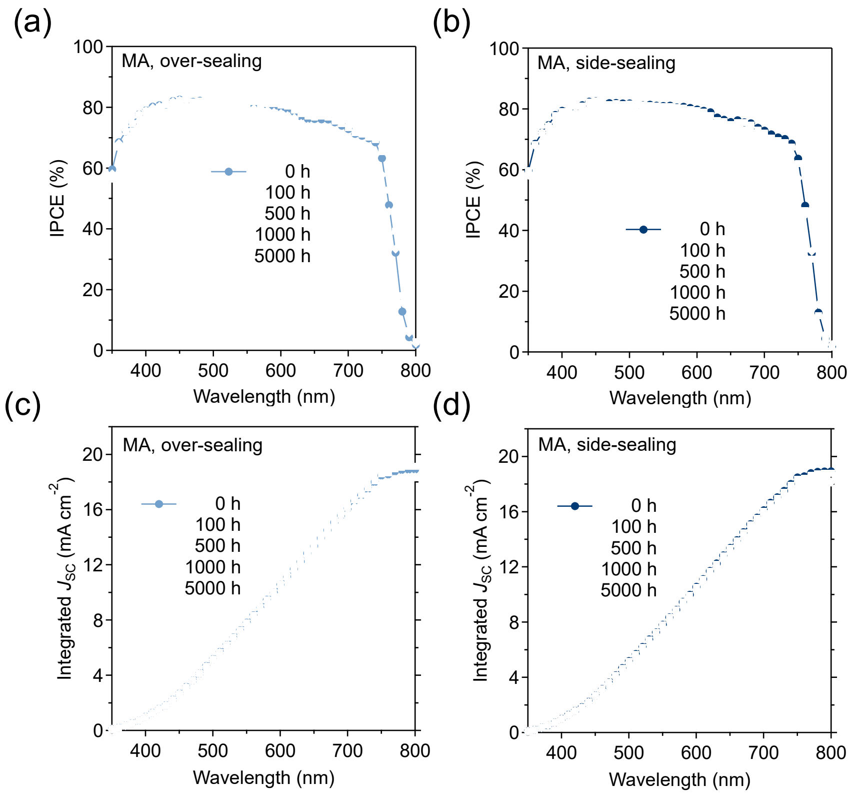

Figure S9. Changes in IPCE spectrum and integrated J_{SC} of MPLE-PSCs with MA perovskite during thermal stability tests. (a, c) over-sealing and (b, d) side-sealing.

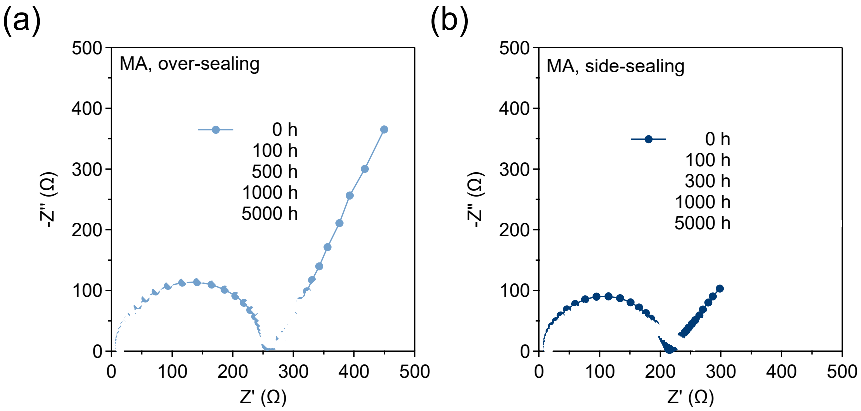

Figure S10. Changes in Nyquist plots of MPLE-PSCs with MA perovskite during thermal stability tests. (a) over-sealing and (b) side-sealing.

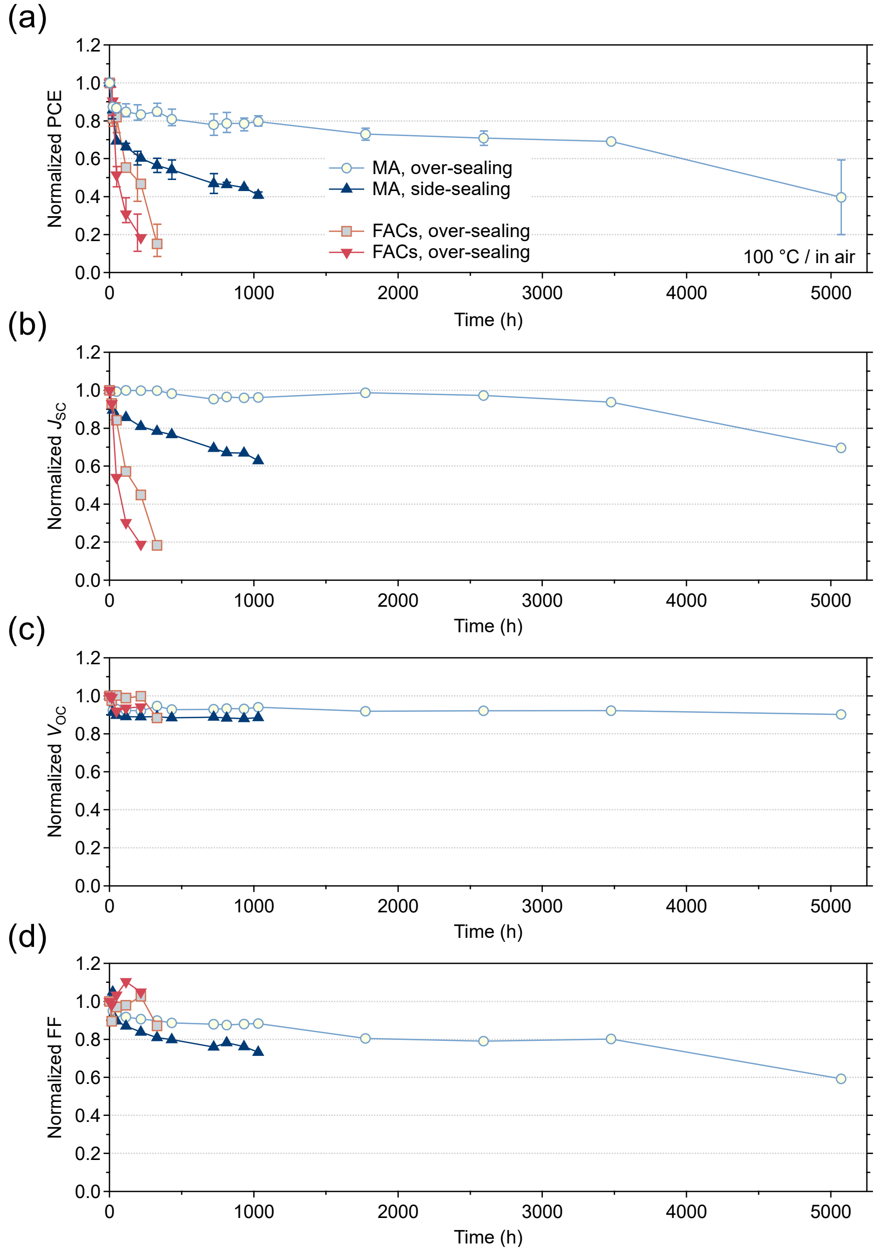

Figure S11. Variations for normalized (a) PCE, (b) J_{SC}, (c) V_{OC}, and (d) FF at the initial value of encapsulated MPLE-PSCs at thermal stability test (100 °C for >5,000 h). The number of used devices was three for the statistical data, and only the average values were plotted in J_{SC}, V_{OC}, and FF.

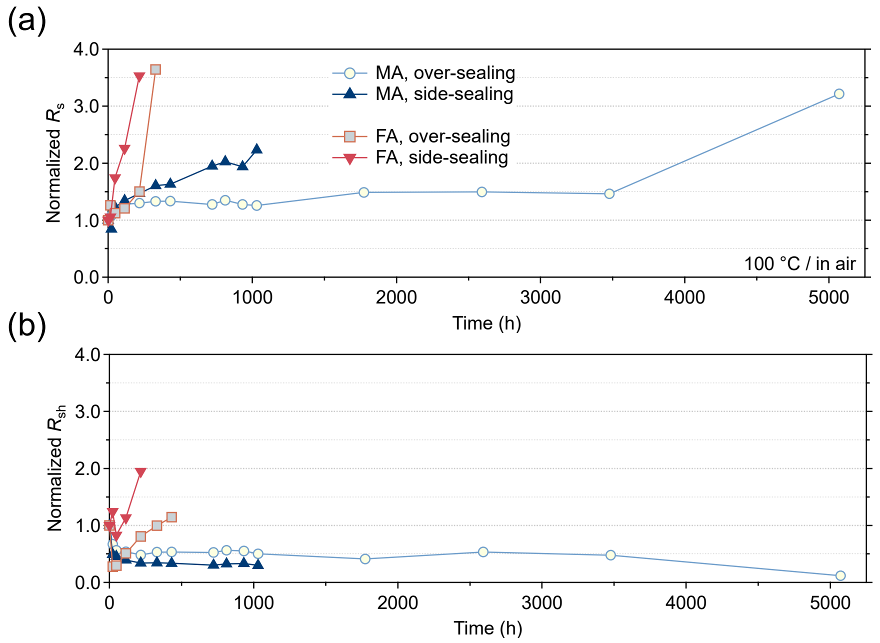

Figure S12. Variations for normalized (a) R_s and (b) R_{sh} at the initial value of encapsulated MPLE-PSCs at thermal stability test (100 °C for >5,000 h). The number of used devices was three for the statistical data, and only the average values were plotted.

Table S5. The time when each device's performance has degraded to 80% of its initial value (T_{80} lifetime) in a thermal stability test at 100 °C. The average times are shown. The number of used devices was three for the statistical data.

light absorber	over-sealing	side-sealing
MA	432 h	22 h
FACs	48 h	15 h

References

[51] Baikie, T.; Fang, Y.; Kadro, J. M.; Schreyer, M.; Wei, F.; Mhaisalkar, S. G.; Graetzel, M.; White, T. J. Synthesis and crystal chemistry of the hybrid perovskite $(CH_3NH_3)PbI_3$ for solid-state sensitised solar cell applications. *J. Mater. Chem. A* **2013**, *1*, 5628. doi:10.1039/c3ta10518k.
